# An Empathy Evaluation System Using Spectrogram Image Features of Audio

**DOI:** 10.3390/s21217111

**Published:** 2021-10-26

**Authors:** Jing Zhang, Xingyu Wen, Ayoung Cho, Mincheol Whang

**Affiliations:** 1 Department of Emotion Engineering, University of Sangmyung, Seoul 03016, Korea; 201934154@sangmyung.kr (J.Z.); 201934153@sangmyung.kr (X.W.); joa6391@gmail.com (A.C.); 2 Department of Human Centered Artificial Intelligence, University of Sangmyung, Seoul 03016, Korea

**Keywords:** empathy evaluation, audio processing, MFCC, machine learning

## Abstract

Watching videos online has become part of a relaxed lifestyle. The music in videos has a sensitive influence on human emotions, perception, and imaginations, which can make people feel relaxed or sad, and so on. Therefore, it is particularly important for people who make advertising videos to understand the relationship between the physical elements of music and empathy characteristics. The purpose of this paper is to analyze the music features in an advertising video and extract the music features that make people empathize. This paper combines both methods of the power spectrum of MFCC and image RGB analysis to find the audio feature vector. In spectral analysis, the eigenvectors obtained in the analysis process range from blue (low range) to green (medium range) to red (high range). The machine learning random forest classifier is used to classify the data obtained by machine learning, and the trained model is used to monitor the development of an advertisement empathy system in real time. The result is that the optimal model is obtained with the training accuracy result of 99.173% and a test accuracy of 86.171%, which can be deemed as correct by comparing the three models of audio feature value analysis. The contribution of this study can be summarized as follows: (1) the low-frequency and high-amplitude audio in the video is more likely to resonate than the high-frequency and high-amplitude audio; (2) it is found that frequency and audio amplitude are important attributes for describing waveforms by observing the characteristics of the machine learning classifier; (3) a new audio extraction method is proposed to induce human empathy. That is, the feature value extracted by the method of spectrogram image features of audio has the most ability to arouse human empathy.

## 1. Introduction

Empathy, a phenomenon of characterizing our understanding and sharing of others’ feelings, is vital to our everyday communication and survival in a social environment [1]. Empathy evaluation has many methods but can be mainly classified into three categories: subjective size evaluation, image processing, and bio-signals [2]. In image processing, a four-camera vision system was established to sample a target from different perspectives. In this system, a global calibration technique was deployed to correlate each individual system. After that, the local point clouds were extracted, filtered, and stitched [3]. The subjective evaluation method usually uses a questionnaire but has limitations due to subjective ambiguity and individual differences. In order to compensate for its limitations, researchers also use objective methods to evaluate empathy.

Immersive environments can induce emotional changes capable of generating states of empathy. Considering an immersive environment as a socio-technical system, human and non-human elements could interact and could also establish strong relationships [4]. In video content, audio sensitively affects human emotions, perceptions, and imagination [5], which can relieve tension or create the feeling of sadness. Therefore, it is important for video creators, such as advertising designers, to understand the relationship between the physical elements of audio and humans [6]. Advertisement videos provide information of products to viewers through various media such as the Internet, on the air, and cable [7]. These videos capture the interest of viewers and increase the purchasing power of products through this empathy. The video content needs to be considered by determining the required empathetic response of the viewer to the content of the video [8]. Whether or not the viewer would empathize with the video content, such as video advertisements, and the judgment of this empathy depends on individual subjective evaluation. An objective and scientific approach or evaluation method, which produces an advertisement video that is highly resonant to viewers, is required for the successful production of an advertisement video [9].

Content creators actively use video content to gain public empathy. Video provides an image or audio to the audience. In his book “Audio-visual on the Screen”, Chion proposed that empathetic music participates in the “feeling of the screen” [10], and the intensity and rhythm of the music are both participatory. Through the use of music, the protagonist’s indifference and the character of moving forward alone are shown. He pointed out that empathetic music often has the effect of “not freezing emotions but enhancing emotions” because music has surprising power [11].

In the movie ‘Tenet’, scales with the same key are continuously repeated to increase the tension. Empathetic audio foregrounds are artifices that play a performative role [12]. Empathetic music is frequently linked to noise; for example, “the shower water that continues to run after Marion’s death in Hitchcock’s Psycho” [13] is discussed as empathetic in the same way as any music cue because the “sonic process continues” without regard for the death that has occurred [14,15].

The foundation of an audio analysis system is feature vector extraction. This paper studies the method of audio signal classification processing. The mainstream method is used to represent three feature sets of time texture, rhythm content, and pitch content. Tzanetakis, G, used the performance and relative importance of the proposed features investigated by training statistical pattern-recognition classifiers using real-world audio collections [16]. Saunders [17] highlighted the difference between music and speech, which attracted considerable attention. In the work of Scheirer and Slaney [18] the average zero-crossing rate and simple threshold of energy characteristics were used, where multiple features and statistical pattern-recognition classifiers are carefully evaluated. Some methods use cepstrum coefficients and hidden Markov models (HMM), and audio signals are divided and classified into “music”, “speech”, “laughter”, and non-speech audio [19].

In addition, the methods of analyzing audio by spectrogram include a gray spectrogram and an RGB spectrogram. A grayscale spectrogram is characterized by quantizing the dynamic range into different regions, and each region is mapped into a monochrome image. An RGB spectrogram is an extension of the pseudo-color mapping process, in which gray intensity is quantized into red, green, and blue (RGB) monochromatic components. These two methods can express the feature mapping of audio as a monochrome image, which is a non-linear mapping function [20].

The purpose of this paper is to determine the degree of empathy in advertisements through audio. There are many ways to analyze audio. This paper combines the power spectrum of MFCC and the application of image RGB to find the audio feature vector for real-time monitoring of empathy in advertisements. The machine learning SVM classifier, which developes a real-time monitoring advertising empathy system, uses the model obtained from the database for classification learning. The feature vector we obtained during the analysis is blue (low range) to green (mid-range) to yellow (upper range) in the spectrum.

An audio image is achieved that can be viewed on a spectrogram. The spectrum is “the intensity map of short-time Fourier transform (STFT) amplitude”. STFT is just a fast Fourier transform sequence of windowed data segments, which usually allows windows to overlap in time [21]. Spectrum is an important representation of audio data.

By analyzing the obtained feature we determine that feature values are low power, medium power, and high power in the high-frequency domain and low power, medium power, and high power in the middle-frequency domain. Moreover, it is low power and high power in the low frequency that can distinguish empathy.

Six classifiers of machine learning methods were used to learn and classify the collected feature values for getting the highest accuracy rate: boosting-tree classifier, decision tree classifier, MLP (multilayer perceptron), random forest classifier, KNN, and SVM. As a result, the correct training rates of the random forest classifier are 99.173% and 86.171%, respectively. However, the highest rate is obtained from the RGB spectrogram filtered by MFCC, a hybrid model of the three feature extraction methods. It can be seen from the results that the empathy ability also gradually increases as the power increases. However, the ability to discriminate empathy characteristics gradually weakens as the frequency domain increases.

The contributions of this study can be summarized as follows: (1) compared with high-frequency and high-amplitude audios, low-frequency, and high-amplitude audios are more likely to make people empathize with the video; (2) by observing the characteristics of the machine learning classifier, it is found that the frequency and amplitude of the audio are important attributes to describe the wave; (3) a new audio extraction method is proposed to induce human empathy. That is, feature values extracted by the method of spectrogram image features of audio has the most ability to arouse human empathy. All the codes are saved in this link: https://github.com/zjing13cc/audio-processing (accessed on 13 October 2021).

## 2. Materials and Methods

### 2.1. Experiment Procedure

We conducted two experiments for the content of this research, namely experiment one in which there are 30 subjects watching 24 advertisements and experiment two with 77 subjects and 8 movie clips.

#### 2.1.1. Subject

There are 30 subjects (15 males and 15 females) who are aged 20–30 years in experiment one, and 77 subjects (38 males and 39 females) aged 20–40 years old in experiment two. All the subjects were asked to have adequate sleep and no caffeine before the experiment and were paid $20 for participation in the whole experimental process. The study was conducted in accordance with the Declaration of Helsinki, and the protocol was approved by the Ethics Committee of Sangmyung University, Seoul, Korea (BE2018-35).

#### 2.1.2. Stimuli

**Figure 1:**
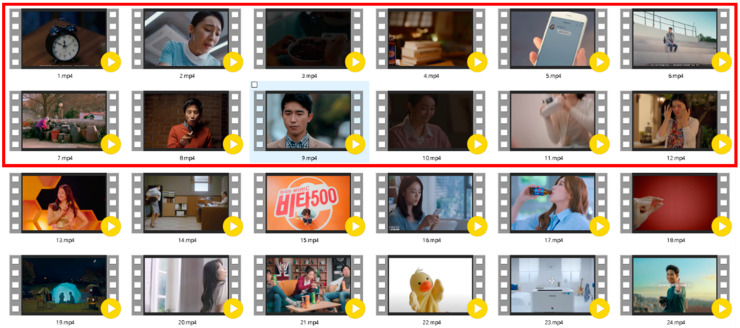
Picture diagrams of 12 empathetic and 12 non-empathetic advertisements in experiment one.

To create an emotional stimulus and induce empathy, this study chose a new stimulus specially designed for this research. According to different emotions, the degree of empathy may vary. In previous research on empathy, media content that triggers emotions can cause empathy more than media content that attracts rationality, and storytelling skills can cause empathy more than enumerating facts or statistical evidence [22,23,24]. Therefore, this study collected YouTube video clips that content providers hoped would convey emotion through advertisements, to induce empathy and content. On the assumption that the audience’s empathy and the effects of the advertising would be high, the researcher selected 50 advertisements with high reviews, high ratings, and high actual sales, and six subjects were investigated in preliminary experiments. Following this, 24 online videos were screened during the first experiment. Regarding the number of times that the same product’s advertisement was played at different times, 24 advertisements were selected from Baxter, Vita 500, Samsung Printer (MLT-D 115), and other products, which are shown in Figure 1. Videos marked with a red box are empathetic, and vice versa are non-empathetic.

**Figure 2:**
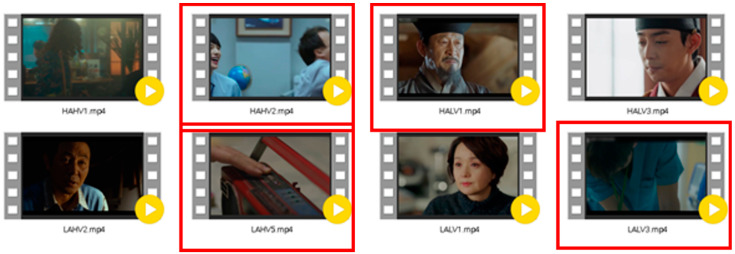
Picture diagrams of 4 empathetic and 4 non-empathetic advertisements in experiment two.

The plots of TV dramas or movies trigger empathy more easily. In order to dig deeper into empathetic elements, we screened the best 8 TV shows and movie clips with high scores in subjective appraisals from 20 of them. They came from clips in Korean TV dramas or movies, as seen in Figure 2. Videos marked with a red box are empathetic videos, and vice versa are non-empathetic videos.

### 2.2. Feature Analysis

This paper used three methods to analyze the emotion of audio: (1) using the MFCC of the human ear pattern filter to extract feature values; (2) using the spectrogram image feature method without pre-processing; and (3) method of combining the above two methods. By comparing the characteristic values extracted by the three methods on empathetic audio and non-empathetic audio shown in Figure 14, the consistent and optimal solution was obtained in the result. The highest training accuracy rate was obtained using machine learning methods.

#### 2.2.1. Mel-Frequency Cepstral Coefficients (MFCCs) Method

The MFCC (mel-frequency cepstral coefficient) is an algorithm that characterizes voice data. You can think of mel as a value derived from the human cochlea. Humans perceive audio through the cochlea. Although the cochlea is curled each part of the cochlea senses different frequencies (frequency) when it is stretched out. Based on the frequency detected by the cochlea humans perceive audio, hence this frequency is used as a feature. However, Cochleas have special properties. The frequency change is well detected in the lower frequency band compared to the higher frequency band. The part of the cochlea is thick when detecting the low-frequency band. However, it becomes thinner when it detects the high-frequency band. Rather than just using the frequency as a feature vector, it would be a more effective way to select features that match the characteristics of the cochlea. A standard set of features for music information are described in the retrieval literature [25,26], including low-level features (RMS level, Bark bands), spectral features (energy in low/middle/high frequencies, spectral centroid, spectral spread, spectral skewness, spectral kurtosis, spectral roll off, spectral crest, spectral flux, spectral complexity), timbral features (mel-frequency cepstral coefficients, t ristimulus), and melodic features (pitch, pitch salience and confidence computed with the YIN algorithm [27], inharmonicity, dissonance) [28,29,30,31] and are shown in Table 1.

**Figure 3:**
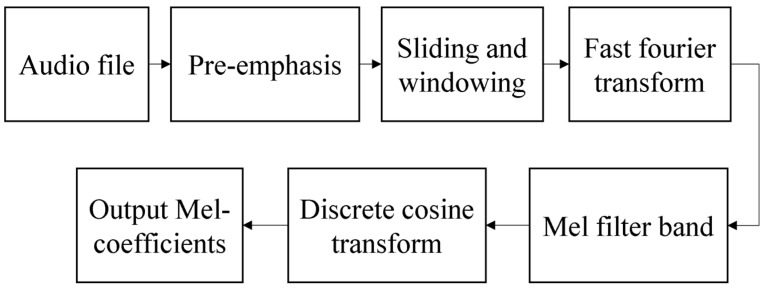
Flow chart of an MFCC feature extraction method.

The quantitative evaluation method of empathy is to analyze the original data in the frequency domain by using the extracted audio features. The power spectrum is extracted from the frequency domain and the frequency components are extracted from a plurality of frequency bands having different frequencies in the frequency spectrum. A single color is then applied to the signals from the plurality of bands to convert the signals into RGB image data; the RGB image data is then stored as learning data, generating a model file, including weights, trained by learning using the learning data. For comparative voice data extracted separately from the input video, the convolutional neural network technology is applied using the training weights of the above model files to compare whether the video is empathetic or uninteresting. We apply a triangular filter at this stage, extracting frequency components from multiple bands with different frequencies on the above power spectrum, and we can apply discrete cosine transform (DCT) to the frequency components of multiple bands [32]. The flow of an MFCC feature extraction method is shown in Figure 3.

**Figure 4:**
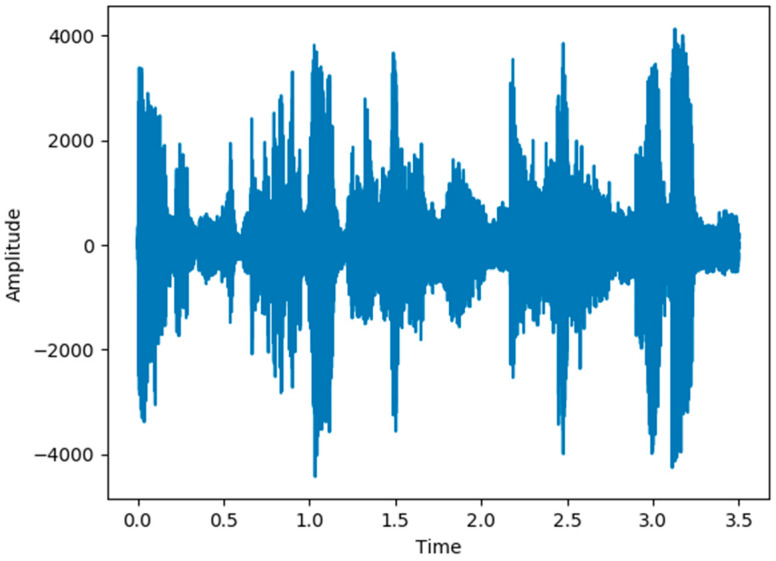
Raw signal in the time domain.

**Figure 5:**
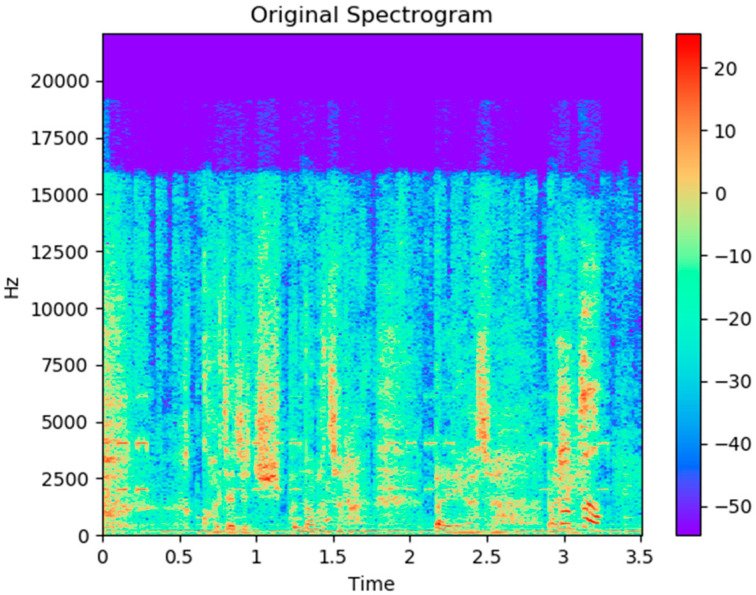
Raw signal in the frequency domain.

In the original data phase, the sampling rate of the input audio signal is usually 30 milliseconds. It is shown with the time domain and frequency domain in Figure 4 and Figure 5.

The pre-emphasis filter is useful in many ways, and it can be applied to the signal x using the first-order filter in the following equation.

y(t) = x(t) − αx(t − 1).(1)

**Figure 6:**
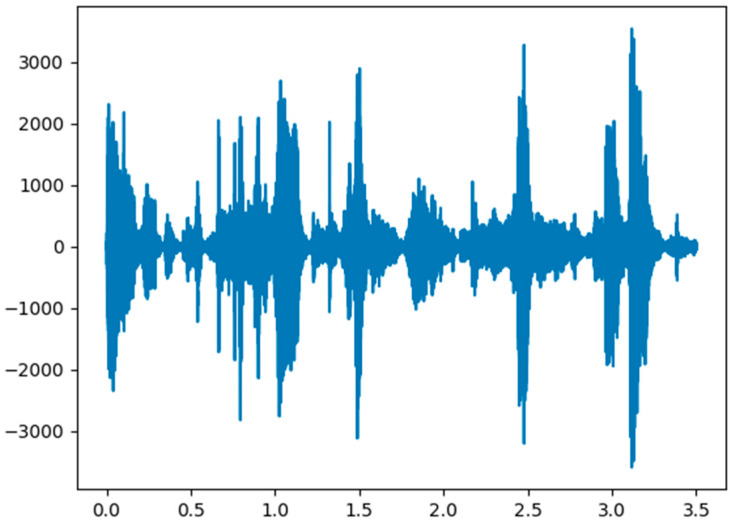
Audio signal in the time domain after the pre-emphasis filter process.

**Figure 7:**
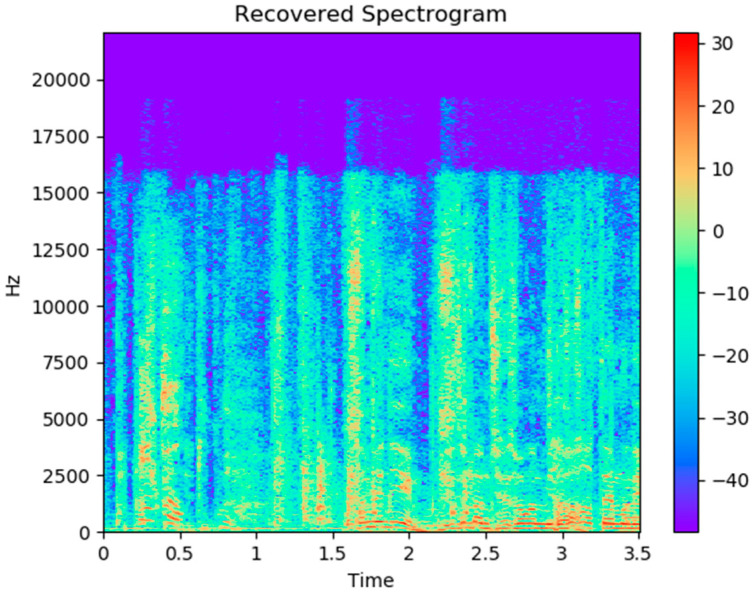
Audio signal in the frequency domain after the pre-emphasis filter process.

The previous work has found that typical values of the filter coefficient (α) is 0.95 or 0.97 [33]. The coefficient is selected as 0.97 in this audio processing. Figure 6 and Figure 7 show the time domain and frequency domain signals after pre-emphasis filtering.

**Figure 8:**
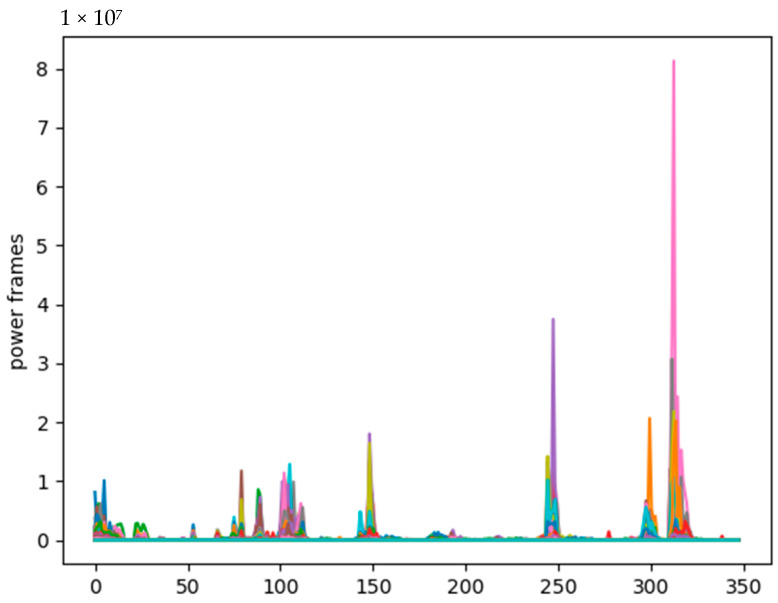
The power spectrum (periodogram) of audio signal was computed after the STFT process.

In the second stage, the fast Fourier transform of the neural network points is performed on each frame to calculate the frequency spectrum, also called short-time Fourier transform (STFT), in which the neural network is usually 256 or 512 [34]. We set NFFT as 512, and then calculate the power spectrum (periodogram) using the following equation in the process. When the number of operation points is an integer power of 2, let x(i) be a finite-length sequence of N points, that is, if there is a value within 0 ≤ n ≤ N − 1, then define x(i) with N points. The x_i_ is the frame of the x-signal as shown in Figure 8, after executing this process, the result can be obtained.

(2) $$P={\left|FFT_{x_{i}}\right|}^{2NP}={\left|FFT_{x_{i}}\right|}^{2N}$$

**Figure 9:**
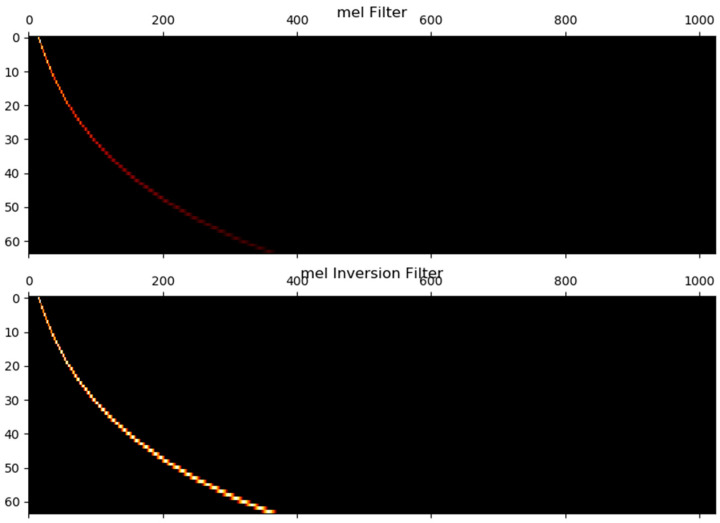
The shape of the mel filter and mel inversion filter.

The last step in calculating the filter bank is to apply a triangular filter to the power spectrum. The mel scale simulates the perception of non-linear human ears to audio as audio is more recognizable to human ears at lower frequencies than at higher frequencies. We can switch between hertz (f) and mel (m) using the following equation. The mel filter and mel inverse filter are shown in Figure 9.

m = 2595log10 (1 + f700),(3)

f = 700(10 m/2595 − 1).(4)

**Figure 10:**
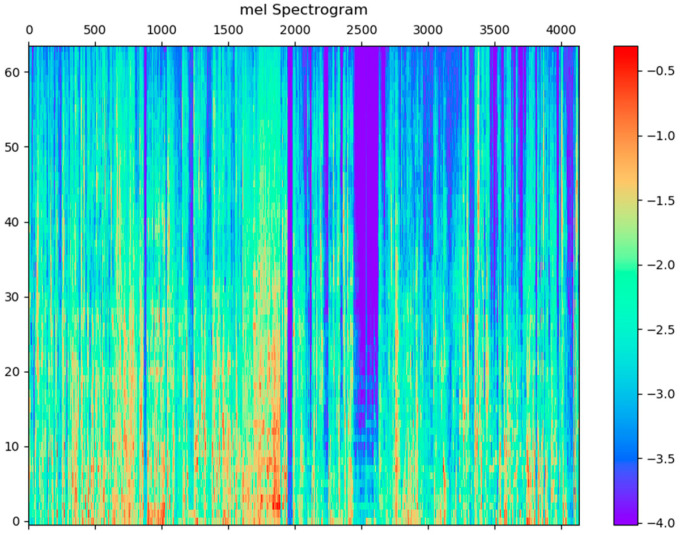
The mel spectrogram after the DCT process.

The discrete cosine transform modifies the filter bank coefficients and generates a compressed representation of the filter banks. Typically, the resulting cepstral coefficients 2–13 are retained and the rest are discarded for automatic speech recognition (ASR). Here 12 values are chosen for num ceps. The mel spectrum after this discrete cosine transform process is shown in Figure 10.

In this paper, audio files are extracted from the advertisement, and then the frequency spectrum of the audio file is obtained using the MFCC. When calculating the power spectral density on the decibel power scale, the width of the Hamming window is 4.15 s, and the size of the sliding window is 50 ms. The median size of the audio signal spectrogram is 371 × 501 pixels.

#### 2.2.2. Spectrogram RGB Image Feature Method

Analysis is carried out by transforming the sampling rate in short blocks of audio of 1 s duration and then calculating the RGB images of spectrograms. These images are used to fine tune the classification of the pre-trained image.

**Figure 11:**
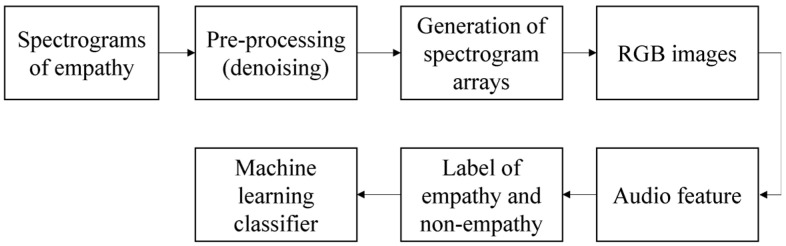
The flow chart of the second algorithm.

The representation of spectrogram on four different frequency scales, linearity, melody, equivalent rectangular bandwidth (ERB), and logarithm, allows the effects of high, medium, and low-frequency characteristics of the audio to be observed separately. The use of red (R), green (G), or blue (B) components of RGB images showed the importance of high, medium, and low-amplitude audio components, respectively. The algorithm is shown in Figure 11.

**Figure 12:**
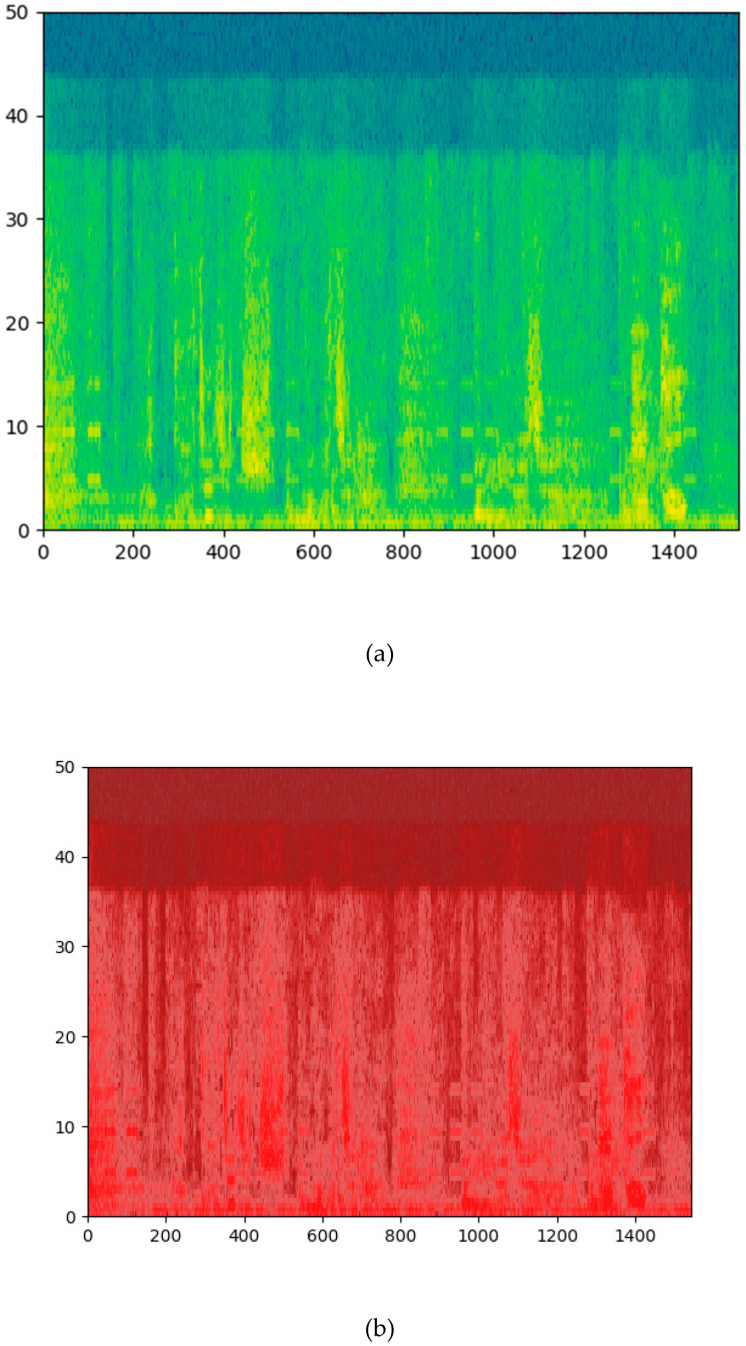

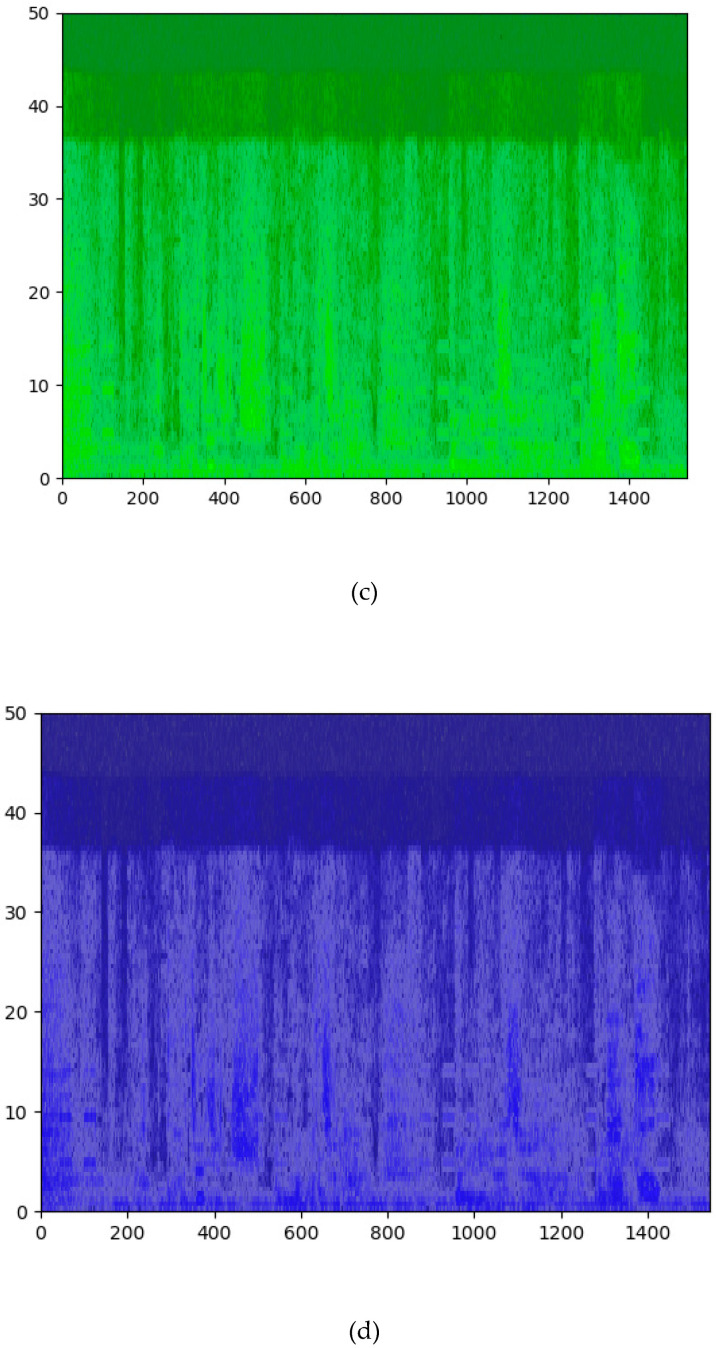
The spectrogram color map of audio has been split into the RGB images. (**a**) The default color picture of MFCC; The RGB pictures from blue (**d**) to green (**c**) to red (**b**).

The algorithm is a method to analyze the color mapping of three “standard” spectrograms in order to find a good representation method of the sample [35]. The first picture is the default color picture of MFCC, ranging from blue (low range) to green (middle range) to red (high range), grayscale is a sequential grayscale mapping from black (low range) to gray (middle range) to white (high range), and finally, Stolar, M.N. et al. [36]. The RGB pictures are uniform sequential color pictures from blue (d) to green (c) to red (b). They are divided into the images shown in Figure 12.

#### 2.2.3. MFCCs and the Spectrogram RGB Image Feature Method

In the first step, we used MFCC to extract the frequency spectrum of the audio file of the video clip (such as advertisements) with a certain sampling rate. The sampling rate, Hamming window width, and sliding size are 30 ms, 4.15 s, and 50 ms respectively, and the output spectral density is calculated from the DB power scale. The median size of the spectrum is 371 × 501 pixels.

The second step is to balance the frequency spectrum (remove noise). At this stage, the frequency spectrum is balanced, which means applying pre-emphasizing filters to the signal to amplify the high frequencies. The pre-processing filter balances the spectrum because the high frequency is smaller than the low frequency according to ear recognition.

The third step is the NN-point FFT calculation. NFFT (number of segments of fast Fourier transform) is 512, and the power spectrum can be calculated by the following formula: 15 degrees represents the results after executing this process. The five steps of applying a triangle filter to the power spectrum in the calculation of a filter bank are to extract the frequency band by applying a triangular mel-scale filter bank (usually 40 filters, n = 40) to the power spectrum. The mel Scale aims to mimic non-linear human ear recognition, in which audio becomes more discriminative at lower frequencies than that at higher frequencies. We can switch between hertz (f) and mel (m) by using the following formula. Therefore, a discrete cosine transform can be applied to decorate the coefficients of the filter bank, and the filter bank can be represented comprehensively. The last step is to transform (calculate) the spectrum with an RGB image. Spectral representations of the three frequency ranges are converted to RGB images to observe the effects of high-, medium-, and low-frequency characteristics. For example, the high-frequency band (15,000–22,500 Hz) is red, the middle-frequency band (7500 Hz) is green, and the low-frequency band (0–7500 Hz) is blue. Using red (r), green (g), or blue (b) components in RGB images, the importance of audio components with high, medium, and low amplitude levels, respectively, are calculated as acoustic characteristics.

**Figure 13:**
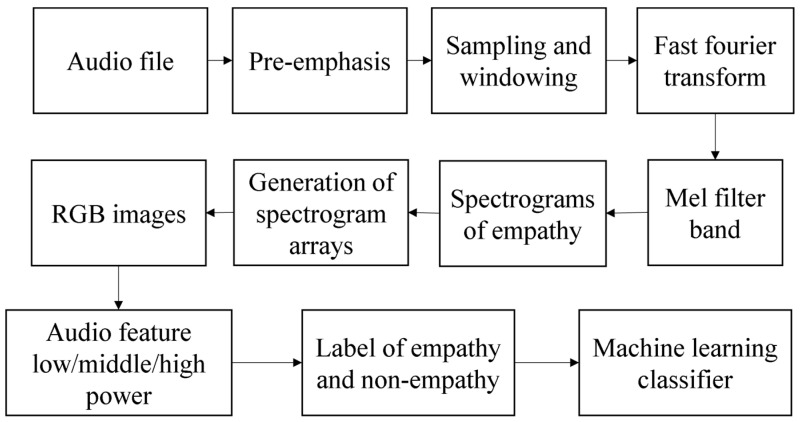
The flow chart of the third algorithm.

In the empathy-evaluation method, using image features according to practical examples, the above-mentioned audio features may include the following: low power, middle power, and high power. The above-mentioned video element and audio attributes are used as learning data. The flow chart of this algorithm is shown in Figure 13. 

#### 2.2.4. Machine Learning Classifier

We tried many classification methods and analyzed their advantages and disadvantages. The classifiers selected here are boosting tree classifier, decision tree classifier, MLP (multilayer perceptron), random forest classifier, KNN (K-nearest neighbor), and SVM (support vector machine) [36,37,38,39,40,41]. We have used 10-fold cross validation and standard parameters for each algorithm. The parameters used are specified in Section 3.2.2.

The boosting tree model uses an additive model (a linear combination of basic functions) and a forward step-by-step algorithm. At the same time, the basic functions use a decision tree algorithm, in which a binary classification tree is used for classification problems, and a binary regression tree is used for regression problems.

Random forest is a meta-estimator, which is suitable for multiple decision tree classifiers on each sub-sample of the data set. It uses the average value to improve prediction accuracy and control overfitting. The sub-sample size is always the same as the original input sample size but if bootstrap is true (the default value) replacement will be used to draw the sample. The performance (function) measures the quality of the split. The supported standards are “gini” for Gini impurity and “entropy” for information gain.

Decision tree learning uses a top-down recursive method, and its basic idea is to construct a tree with the fastest decrease in entropy as a measure of information entropy. The entropy value of the leaf node is zero and the instances in each leaf node belong to the same class currently. The most important advantage of the decision tree algorithm is that it can be self learning. In the learning process, the user need not have much background knowledge but can only label the training examples in a better way. The key to establishing the decision tree is to determine which attribute is selected as the classification basis in the current state. According to different objective functions, there are three main algorithms for building a decision tree: ID3, C4.5, and CART.

The support vector machine (SVM) is non-linear mapping. It uses the inner product kernel function to replace the non-linear mapping in the high-dimensional space. The goal of SVM is to divide the optimal hyperplane of feature space, and the idea of maximizing the classification margin is the core of the SVM method. The SVM learning problem can be expressed as a convex optimization problem so that the known effective algorithms can be used to find the global minimum of the objective function. For this method, the generalization error rate is low, the classification speed is fast, and the results are easy to interpret. However, the disadvantage is that the SVM algorithm is difficult to implement for large-scale training samples as it is sensitive to missing data, selections of parameters, and kernel functions, and the “dimensional disaster” has to be avoided. However, for large-scale training samples, the SVM algorithm is very difficult to implement.

The KNN algorithm is a simple and robust classification technology. Test feature vectors are classified by looking for the k nearest neighbor vectors. The distance between the training vector and the test vector is calculated by measuring the difference in measurement technique [42]. Its training time is not very complicated, except that it has very high precision with no assumption data. The disadvantage is the large amount of calculation, the non-balanced sample, and that it requires a large amount of memory. The KNN’s algorithm classifier will be more suitable for the feature data of the spectrogram extracted from the audio.

## 3. Results

### 3.1. Statistical Analysis Result

#### 3.1.1. Mel-Frequency Cepstral Coefficients (MFCCs) Result

**Figure 14:**
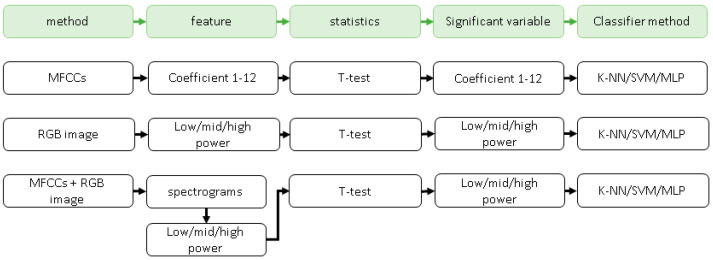
The flow chart of the three methods for empathy evaluation in audio signal processing.

The assumption we have made is that the extracted audio feature can distinguish between empathetic and non-empathetic videos. The first method extracted the audio features in the video that are the independent variables along with its sound features shown in Figure 14. The mel-frequency cepstral coefficients (MFCCs) of a signal are a small set of features that concisely describe the overall shape of a spectral envelope. Eigenvalues were extracted for *t*-test statistical analysis, which tested the two previous hypotheses and were carried out by adjusting the α levels of each test by 0.05. Results showed that empathy and non-empathy were mainly influenced by the healthy body elements (coefficients F1, F2, F3, F4, F5, F6, F7, F8, F10) between the two groups (*p* < 0.001). The differences obtained from the data are shown in Table 2 and Table 3.

#### 3.1.2. Spectrogram RGB Image Feature Result

The second method of audio extraction is extracting spectrogram RGB images of audio features. Audio characteristics obtained by this method are low power, medium power, high power, low frequency, middle frequency, and high frequency. The results show that empathy or non-empathy of the two groups were mainly influenced by the healthy physical characteristics (high power mean, medium power mean, and low power mean) (*p* < 0.001). The two differences obtained from the data are shown in Table 4 and Table 5.

#### 3.1.3. MFCCs and Spectrogram RGB Image Feature Result

Finally, the third method is a combination of the first two methods. The difference between videos is whether they are the main reason for empathy or not. Using the MFCC method (with a similar structure of the human ear vortex) and the power spectrum of RGB extracts with feature values such as low power, middle power, high power, low frequency, middle frequency, high frequency, the results indicated that empathy or non-empathy of the two groups of participants was mainly affected by the sound and physical elements featured (low-power mean, low-power std, middle-power mean, middle-power std, high-power std) (*p* < 0.001) deviation) (*p* < 0.001). The differences obtained from the data are shown in the Table 6 and Table 7.

**Figure 15:**
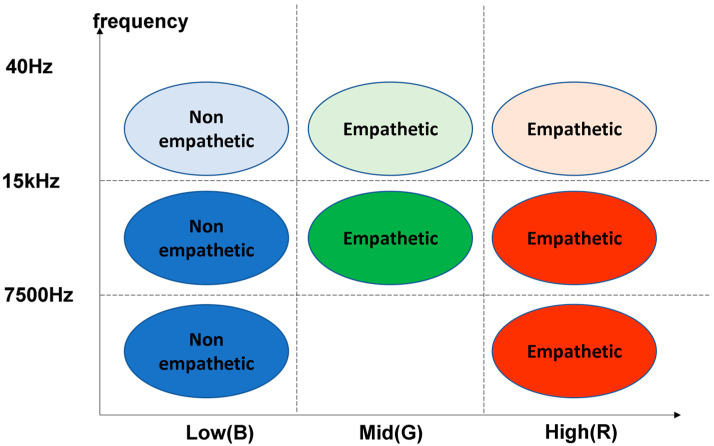
The flow chart of three methods for empathy evaluation in audio signal processing.

According to the statistical analysis results of eigenvalues obtained by the three methods, it is found that in the low-frequency domain it is easier to induce human empathy as the audio power increases. The average error number of the group without the empathy label was significantly higher (m = 2371.9, SD = 29.2; *p* < 0.001) than that of the empathy group (m = 1587.4, SD = 25.8; *p* = 0.001) in the system. As the frequency increases, the ability to induce empathy gradually weakens, such as the value of low power means of empathy label and high-power means of the empathy label: the average number of errors in non-empathy label group is significantly higher (m = 1587.4, SD = 25.8; *p* < 0.001) was higher than that of empathetic group (m = 1.48, SD = 0.09; *p* < 0.001). The result of extracting features in low power and high power was the difference between the empathetic and non-empathetic label in experiment one. The results of experiment two also have the same trend as experiment one in the empathetic and non-empathetic labels as shown in Figure 15.

### 3.2. Classification for All Methods Result

In the mixed model of RGB image scale features and MFCC spectrogram (the RGB scale spectrogram and MFCC spectrogram are given in Table 5), the audio feature values are extracted respectively, and are statistically analyzed. Finally, the meaningful feature value is used for classification by the machine learning classifier.

#### 3.2.1. Multi-Conditional Training

The classifiers selected here are boosting tree classifier, decision tree classifier, MLP (multilayer perceptron), random forest classifier, KNN (K-NearestNeighbor), and SVM (support vector machine). You can see from the results in Table 8 that for the characteristics of the audio signal, the accuracy of the SVM classifier is higher than the other two classifiers, which achieved better predictive value. From the accuracy rate, it can be seen that the mixed model using the MFCC filter and RGB image processing has the highest accuracy rate with 99.173% training data and 86.171% test data, which are better than that of other classifiers: AdaBoost classifier (78.092% training data; 78.125% test data), decision tree (83.824% training data; 81.196% test data), SVM classifier (75.3% training data; 62.3% test data), KNN classifier (71.2% training data; 60.5% test data), and MLP classifier (67.823% training data; 67.9% test data).

#### 3.2.2. Classification and Evaluation

The classifiers selected here are boosting tree classifier, decision tree classifier, MLP (multilayer perceptron), random forest classifier, KNN (K-nearest neighbor), and SVM (support vector machine) [36,37,38,39,40]. We have used 10-fold cross validation and standard parameters for each algorithm. The parameters used are specified in Table 9, Table 10, Table 11, Table 12 and Table 13.

AdaBoost uses an additive model to linearly combine weak classifiers. For example, AdaBoost uses a weighted majority vote to increase the weight of a classifier with a small error rate and reduce the weight of a classifier with a large error rate. The data of this experiment found that the maximum training accuracy rate was 78.092% by changing the learning rate to 0.8 and the test accuracy rate to 78.125%.

Each decision tree of a random forest is a classifier (assuming that it is a classification problem). N trees will have N classification results for an input sample. When the random forest integrates all the classification voting results, the category with the most votes is designated as the final output. From the data results of this experiment, when there are 80 trees the maximum training accuracy rate is 99.173% with 86.171% test accuracy rate.

With the maximum depth that the tree is allowed to grow to, the deeper you allow the more complex the model is. There is a good golden point between too high and too low. Think of maximum_depth as a hyperparameter and use grid/random search with cross-validation to find a good value for the maximum_depth. We found that a maximum depth of 10 is the best training result, when the training accuracy rate is 83.824%, and the test accuracy rate is 81.196%.

For the number of iterations of MLP, the accuracy of the training set and the test set are also different. We chose to iterate from 200 to 500 every 50 steps. The result obtained is that when the audio training set is iterated to 350 times using the MLP classifier, the accuracy rate is no longer obvious. The accuracy rate of the training set and the accuracy rate of the test set is maintained at 67.823% and 67.9%, respectively.

**Figure 16:**
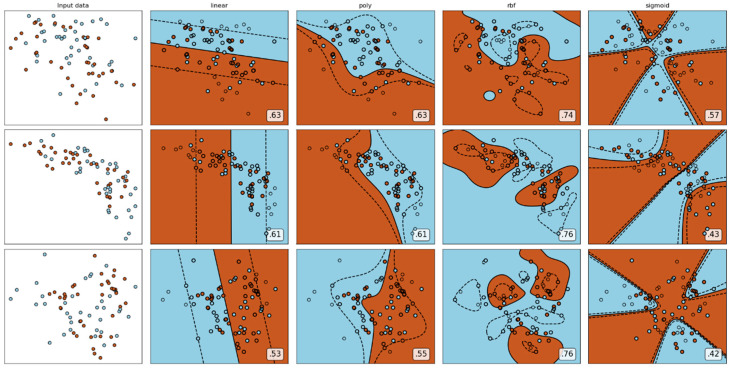
The feature values of the audio signal are used to form three sets of visual images obtained by using different kernel functions.

The advantage of the SVM classifier lies in that it is a kernel function. Generally it uses ‘RBF’, ‘linear’, ‘poly’, etc. As shown in Figure 16, it is found that the function model works best when using ‘RBF’ parameters. Another important parameter is that a larger C is equivalent to punishing the slack variable. It is hoped that the slack variable will be close to 0 and that the penalty for misclassification will increase, which tends to be the case of fully splitting the training set. It is why the generalization ability is weak with a high accuracy of training set. The value of C is small, which reduces the penalty for misclassification, allows fault tolerance, is regarded as noise points, and has strong generalization ability [43,44].

In the machine learning model, the parameters that need to be selected manually are called hyperparameters. The name of ‘GridSearchCV’ can be divided into two parts, ‘GridSearch’ and CV, namely grid search and cross-validation. Both names are very easy to understand. Grid search, searches for parameters within the specified parameter range, adjusts the parameters in sequence according to the step length, uses the adjusted parameters to train the learner, and finds the parameter with the highest accuracy on the verification set from all the parameters. This is a process of training and comparison. GridSearchCV can guarantee to find the most accurate parameter within the specified parameter range, but this is also the flaw of grid search. It requires traversing all possible parameter combinations, which is very time consuming in the face of large data sets and multiple parameters. In this analysis SVM is used for classification, evaluation, and to establish a software-obsolete evaluation model. Firstly, the GridSearchCV class is used to select the optimal gamma C and gamma of the SVM, and the typical C and gamma are selected for the svm.SVC model [44]. The results of the SVM model evaluation are shown in Table 6. From the results of NuSVC, SVC, and LinearSVC in the SVM model, SVC is the best choice in this model. The kernel function of svm.SVC (gamma:1; learning rate: 0.001) is more suitable for audio processing when using the SVM classifier shown in Table 6.

## 4. Conclusions

In this paper, 24 advertisement videos on YouTube and 8 drama video clips are selected as stimuli. The subjects watched the videos and made a subjective assessment on empathy levels. In this paper, the audio signal in the advertisement was mainly used for analysis. There were three analysis methods used: MFCC, RGB spectrogram, and filtered RGB spectrogram of mixed model MFCC. When using the statistical *t*-test, there were differences in audio feature values between the empathy tags and non-empathy tags. We then used six classifiers with higher recognition of machine learning methods to learn and classify the collected feature values, and calculate with of the obtained accuracy rates is the highest: boosting tree classifier, decision tree classifier, MLP (multilayer perceptron), random forest classifier, KNN, and SVM. The correct training rate of the random forest classifier is 99.173% and the test rate of the random forest classifier with 86.171%. The training accuracy and test accuracy of RGB spectrogram filtered by MFCC, a hybrid model of the three feature extraction methods, is the highest.

The method used in statistics is to compare the power in the low frequency domain. As the audio power increases, it is more likely to cause empathetic resonance. As the frequency increases, the result of the ability to induce empathy is discovered. In a word, the contributions of this study are as follows. First, for those who make commercial video, music, and music with low frequency and high amplitude are more likely to resonate with video than audio with high frequency and high amplitude. Secondly, by observing the features of the machine learning classifier, it can be found that frequency and audio amplitude are important attributes to describe waves. Thirdly, a new audio extraction method is proposed to induce human empathy. That is, the feature value extracted by the method of the spectrogram image features of audio has the most ability to arouse human empathy.

## 5. Discussion

The conclusion is that increasing the strength of audio in advertising production enhances the viewers’ empathy in this system. The experiments presented here have demonstrated clear advantages of the proposed methodology by comparing them to traditional methods utilizing arrays of spectrogram magnitudes [45,46,47]. This agrees with Andrei C. Miu, who has said that positive emotions trigger empathy more than negative emotions [48], and Jing Z, etc. the power of positive emotion audio is higher than the power of negative emotion audio [23]. Yoo, S. et al. who thought the effect of a co-viewer may only impact on empathetic responses when participants felt higher emotional intensity [32]. Therefore, it can be concluded that the ability of inducing human empathy would increase with an increase in frequency, e.g., the value of low-power mean of the non-empathetic label and the empathetic label where the average number of errors is significantly higher in the group of non-empathetic labels than those with empathetic labels. As the frequency increases, the ability to induce empathy gradually weakens, e.g., the value of low-power and high-power means of the empathetic label where the average number of errors was significantly higher in the group with the non-empathetic labels than those in the group of empathetic labels.

In the future, the image feature value and the audio feature value in the advertisement would be combined in order to achieve a greater effect on the empathy stimulation of the advertisement.

## Figures and Tables

**Table 1:** Summary of the features of audio.

Spectral	Spectral centroid, spectral roll off, spectral flux, MFCCs.
Temporal	Zero-crossing profile, key clarity, harmonic change, musical mode.
Rhythmic	Beat histogram, average tempo (BPM).

**Table 2:** The average error (coefficients F1, F2, F3, F4, F5, F6, F7, F8, F10) (*p* < 0.001) of the characteristic values of acoustic and physical elements is the difference between empathetic and non-empathetic labels in experiment one.

Label	F1		F2		F3		F4		F5	
	Mean	Std	Mean	Std	Mean	Std	Mean	Std	Mean	Std
Non-empathetic	1.11	0.41	−92.85	0.68	44.63	0.89	−153.48	0.98	107.12	1.16
Empathetic	−30.97	0.68	−127.33	1.16	71.69	1.49	−172.1	1.75	112.66	1.94
**Label**	**F6**		**F7**		**F8**		**F10**			
	**Mean**	**Std**	**Mean**	**Std**	**Mean**	**Std**	**Mean**	**Std**		
Non-empathetic	−198.14	1.2	76.23	1.41	−145.67	1.32	−118.32	1.34		
Empathetic	−240.14	2.12	81.7	2.41	114.56	2.22	−138.58	2.19		

**Table 3:** The average error (coefficients F1, F2, F3, F4, F5, F6, F7, F8, F10) (*p* < 0.001) of the characteristic values of acoustic and physical elements is the difference between empathetic and non-empathetic labels in experiment two.

Label	F1		F2		F3		F4		F5	
	Mean	Std	Mean	Std	Mean	Std	Mean	Std	Mean	Std
Empathetic	−240.02	1.84	133.42	0.55	−32.75	0.42	16.39	0.27	−12.2	0.32
Non-empathetic	−351.08	2.51	121.99	0.78	−13.71	0.91	23.8	0.35	−1.69	0.46
**Label**	**F6**		**F7**		**F8**		**F10**			
	**Mean**	**Std**	**Mean**	**Std**	**Mean**	**Std**	**Mean**	**Std**		
Empathetic	11.9	0.23	−8.47	0.18	−2.52	0.17	−1.87	0.16		
Non-empathetic	5.28	0.2	−1.73	0.21	1.13	0.13	−2.92	0.16		

**Table 4:** Average error (*p* < 0.001) of characteristic values of audio and physical elements (low power, medium power, high power, low frequency, middle frequency, and high frequency) compared with empathetic and non-empathetic labels in experiment one.

Label	R-High		G-High		B-High		R-Middle	
	Mean	Std	Mean	Std	Mean	Std	Mean	Std
Non-empathetic	48.8	0.1	119.1	0.2	137.6	0.07	61.6	0.45
Empathetic	48.1	0.06	118	0.2	137.9	0.03	59	0.35
**Label**	**G-Middle**		**B-Middle**		**R-Low**		**B-Low**	
	**Mean**	**Std**	**Mean**	**Std**	**Mean**	**Std**	**Mean**	**Std**
Non-empathetic	−174.6	0.38	118.7	0.3	98.3	0.4	94.6	0.2
Empathetic	−171.6	0.36	120.6	0.2	96.6	0.4	95.5	0.2

**Table 5:** Average error (*p* < 0.001) of characteristic values of audio and physical elements (low power, medium power, high power, low frequency, middle frequency, and high frequency) compared with empathetic and non-empathetic labels in experiment two.

Label	R-High		G-High		B-High		R-Middle	
	Mean	Std	Mean	Std	Mean	Std	Mean	Std
Non-empathetic	67.1	0.78	119.1	0.2	117.7	0.53	68.3	0.76
Empathetic	60.0	0.62	118.0	0.2	122.3	0.43	61.7	0.6
**Label**	**G-Middle**		**B-Middle**		**R-Low**		**B-Low**	
	**Mean**	**Std**	**Mean**	**Std**	**Mean**	**Std**	**Mean**	**Std**
Non-empathetic	168.5	0.63	116.4	0.51	65.7	0.78	116.7	0.53
Empathetic	164.9	0.52	120.6	0.41	58.6	0.63	121.2	0.43

**Table 6:** Average error of characteristic value of audio and physical element features used for difference comparison between empathetic/non-empathetic labels in experiment one (low-power average, low-power standard, medium-power average, medium-power standard, and high-power means high-power standard) (*p* < 0.001).

Label	Low-Power Mean		Low-Power Std		Middle-Power Mean	
	Mean	Std	Mean	Std	Mean	Std
Non-empathetic	2371.9	29.2	9412.2	123.1	74.87	3.47
Empathetic	1587.4	25.8	6086.1	106.4	92.36	4.97
**Label**	**Middle-Power Std**		**High-Power Mean**		**High-Power Std**	
	**Mean**	**Std**	**Mean**	**Std**	**Mean**	**Std**
Non-empathetic	213.7	10.8	0.86	0.05	4.31	0.33
Empathetic	227.6	12.8	1.48	0.09	7.61	0.47

**Table 7:** Average error of characteristic value of audio and physical element features used for difference comparison between empathetic/non-empathetic labels in experiment two, (low-power average, low-power standard, medium-power average, medium-power standard, and high-power means high-power standard) (*p* < 0.001).

Label	Low-Power Mean		Low-Power Std		Middle-Power Mean	
	Mean	Std	Mean	Std	Mean	Std
Non-empathetic	268.5	3.19	7429.3	71.82	0.067	0.003
Empathetic	133.9	12.67	1531	19.19	0.199	0.007
**Label**	**Middle-Power Std**		**High-Power Mean**		**High-Power Std**	
	**Mean**	**Std**	**Mean**	**Std**	**Mean**	**Std**
Non-empathetic	0.33	0.01	36.22	0.56	237	3.37
Empathetic	0.86	0.03	36.77	0.46	237.5	3.94

**Table 8:** The feature values obtained by the three audio extraction methods are training accuracy and test accuracy after machine learning of the BT, DT, MLP, RF, KNN, and SVM classifiers.

Classifier Method	Training Accuracy	Test Accuracy
Extraction feature of method 1		
AdaBoost	66.284%	54.212%
Decision tree	73.244%	64.956%
Random forest	**76.53%**	**70.791%**
SVM	74%	66.5%
KNN	65.3%	66.2%
MLP	55.3%	55.1%
Extraction feature of method 2		
AdaBoost	72.92%	68.5%
Decision tree	77.842%	72.66%
Random forest	**78.732%**	**72.721%**
SVM	61.2%	58.5%
KNN	70.2%	61.7%
MLP	58.5%	50.5%
Extraction feature of method 3		
AdaBoost	78.092%	78.125%
Decision tree	83.824%	81.196%
Random forest	**99.173%**	**86.171%**
SVM	75.3%	62.3%
KNN	71.2%	60.5%
MLP	67.823%	67.9%.

**Table 9:** The evaluation result of a learning rate of 0.1, 0.4, 0.8 in the AdaBoost model.

Model	Label	Precision	Recall	F1-Score	Support
Learning rate of 0.1	Non-empathetic	0.70	0.78	0.74	2299
	Empathetic	0.75	0.66	0.70	2017
	Avg/total	0.73	0.72	0.72	4316
Learning rate of 0.4	Non-empathetic	0.76	0.80	0.78	2299
	Empathetic	0.78	0.74	0.76	2017
	Avg/total	0.77	0.77	0.77	4316
Learning rate of 0.8	Non-empathetic	0.79	0.77	0.78	2299
	Empathetic	0.77	0.79	0.78	2017
	Avg/total	0.78	0.78	0.78	4316

**Table 10:** The evaluation result of the number of trees being 10, 40, 80 in the random forest model.

Model	Label	Precision	Recall	F1-Score	Support
Number trees of 10	Non-empathetic	0.82	0.88	0.85	2299
	Empathetic	0.87	0.81	0.84	2017
	Avg/total	0.85	0.84	0.84	4316
Number trees of 40	Non-empathetic	0.85	0.87	0.86	2299
	Empathetic	0.87	0.84	0.86	2017
	Avg/total	0.86	0.86	0.86	4316
Number trees of 80	Non-empathetic	0.86	0.87	0.86	2299
	Empathetic	0.87	0.85	0.86	2017
	Avg/total	0.86	0.86	0.86	4316

**Table 11:** The evaluation result of a maximum depth of 10, 50, 100 in the decision tree model.

Model	Label	Precision	Recall	F1-Score	Support
Maximum depth of 10	Non-empathetic	0.82	0.81	0.81	2299
	Empathetic	0.81	0.82	0.81	2017
	Avg/total	0.81	0.81	0.81	4316
Maximum depth of 50	Non-empathetic	0.81	0.82	0.81	2299
	Empathetic	0.81	0.80	0.81	2017
	Avg/total	0.81	0.81	0.81	4316
Maximum depth of 100	Non-empathetic	0.81	0.81	0.81	2299
	Empathetic	0.81	0.80	0.81	2017
	Avg/total	0.81	0.81	0.81	4316

**Table 12:** The evaluation result of the iteration of ordinal numbers 200, 350, 500 in the MLP model.

Model	Label	Precision	Recall	F1-Score	Support
Iteration ordinal number 200	Non-empathetic	0.62	0.93	0.75	2299
	Empathetic	0.86	0.42	0.56	2017
	Avg/total	0.74	0.68	0.65	4316
Iteration ordinal number 350	Non-empathetic	0.62	0.95	0.75	2299
	Empathetic	0.88	0.41	0.56	2017
	Avg/total	0.75	0.68	0.65	4316
Iteration ordinal number 500	Non-empathetic	0.62	0.95	0.75	2299
	Empathetic	0.88	0.41	0.56	2017
	Avg/total	0.75	0.68	0.65	4316

**Table 13:** The evaluation result of NuSVC, SVC, and LinearSVC in the SVM model.

Model		Precision	Recall	F1-Score	Support
svm.NuSVC	0	0.61	0.73	0.67	2299
	1	0.64	0.51	0.57	2017
	Avg/total	0.58	0.52	0.47	4316
svm.SVC	0	**0.77**	**0.77**	**0.72**	2299
	1	**0.71**	**0.75**	**0.67**	2017
	Avg/total	0.74	0.76	0.69	4316
svm.LinearSVC	0	0.66	0.69	0.68	2299
	1	0.66	0.63	0.64	2017
	Avg/total	0.58	0.66	0.66	4316
